# Response to Treatment, Racial and Ethnic Disparity, and Survival in Patients With Breast Cancer Undergoing Neoadjuvant Chemotherapy in the US

**DOI:** 10.1001/jamanetworkopen.2023.5834

**Published:** 2023-03-30

**Authors:** Sarah Shubeck, Fangyuan Zhao, Frederick M. Howard, Olufunmilayo I. Olopade, Dezheng Huo

**Affiliations:** 1 Department of Surgery, The University of Chicago, Chicago, Illinois; 2 Department of Public Health Sciences, The University of Chicago, Chicago, Illinois; 3 Section of Hematology and Oncology, Department of Medicine, The University of Chicago, Chicago, Illinois

## Abstract

**Importance:**

With the increasing delivery of neoadjuvant chemotherapy (NACT) for patients with breast cancer in the US, it is important to know whether there is differential response to NACT by race and ethnicity and the potential long-term outcomes.

**Objective:**

To examine whether there were any racial and ethnic differences in pathologic complete response (pCR) rate following NACT and, if so, whether they varied by molecular subtype and were associated with survival.

**Design, Setting, and Participants:**

A retrospective cohort study was conducted including patients with stage I to III breast cancer diagnosed between January 2010 and December 2017 who underwent surgery and received NACT; median follow-up was 5.8 years, and data analysis was conducted from August 2021 to January 2023. Data were obtained from the National Cancer Data Base, a nationwide, facility-based, oncology data set that captures approximately 70% of all newly diagnosed cases of breast cancer in the US.

**Main Outcomes and Measures:**

Pathologic complete response, defined as ypT0/Tis ypN0, was modeled using logistic regression. Racial and ethnic differences in survival were analyzed using a Weibull accelerated failure time model. Mediation analysis was conducted to measure whether racial and ethnic differences in the pCR rate affect survival.

**Results:**

The study included 107 207 patients (106 587 [99.4%] women), with a mean (SD) age of 53.4 (12.1) years. A total of 5009 patients were Asian or Pacific Islander, 18 417 were non-Hispanic Black, 9724 were Hispanic, and 74 057 were non-Hispanic White. There were significant racial and ethnic differences in pCR rates, but the differences were subtype-specific. In hormone receptor–negative (HR−)/erb-b2 receptor tyrosine kinase 2 (*ERBB2*; formerly *HER2* or *HER2/neu*)*–*positive (*ERBB2*+) subtype, Asian and Pacific Islander patients achieved the highest pCR rate (56.8%), followed by Hispanic (55.2%) and non-Hispanic White (52.3%) patients with the lowest pCR rate seen in Black patients (44.8%). In triple-negative breast cancer, Black patients had a lower pCR rate (27.3%) than other racial and ethnic groups (all >30%). In HR+/*ERBB2*− subtype, Black patients had a higher pCR rate (11.3%) than other racial/ethnic groups (all ≤10%). In mediation analysis, racial and ethnic differences in achieving pCR after NACT could explain approximately 20% to 53% of the subtype-specific survival differences across racial and ethnic groups.

**Conclusions and Relevance:**

In this cohort study of patients with breast cancer receiving NACT, Black patients had a lower pCR rate for triple-negative and HR−/*ERBB2*+ breast cancer but a higher pCR rate for HR+/*ERBB2*− diseases, whereas Asian and Pacific Islander patients had a higher pCR rate for HR−/*ERBB2*+ diseases. Tumor grade and *ERBB2* copy number could account for some of these within-subtype disparities, but further studies are warranted. Inability to achieve a pCR can mediate in part, but not entirely, the worse survival outcomes experienced by Black patients.

## Key Points

**Question:**

Are there racial and ethnic differences in response to neoadjuvant chemotherapy of breast cancer that may have survival implications?

**Findings:**

In this cohort study of 107 207 patients with stage I to III breast cancer, significant racial and ethnic differences in pathologic complete response rates were observed. These racial and ethnic differences were subtype-specific and were found to account for a substantial portion of the identified survival disparity.

**Meaning:**

The findings of this study suggest that biomarker-informed optimal treatment could improve treatment response, and in turn, may ultimately reduce racial and ethnic disparity in survival outcomes.

## Teaser

This cohort study examines discrepancies in response to neoadjuvant chemotherapy among patients in differing racial and ethnic groups with breast cancer.

## Introduction

Neoadjuvant chemotherapy (NACT) was initially used to treat inoperable, locally advanced breast cancer.^1^ However, in contemporary strategies there are more indications for the use of initial medical therapy. This includes using NACT to reduce the size of breast tumors, decrease the incidence of positive nodes, and provide guidance for additional adjuvant therapy.^1,2^ When patients achieve a pathologic complete response (pCR) to NACT, they benefit from improved oncologic outcomes including rates of recurrence and survival.^1,2,3^ The rate of pCR has been demonstrated to correlate with biologic subtype and often affects further treatment decisions, including the scale of surgical approach and potential indications for adjuvant therapy.^4^

While substantial improvements in breast cancer survival have been achieved with modern treatment regimens, Black women continue to experience worse survival outcomes.^5^ However, when receipt of treatment is standardized in the context of clinical trials, often the pCR rate and oncologic outcomes have not differed substantially by race and ethnicity.^6,7^ Racial grouping in the US is a social construct and in the clinical practice setting, patients categorized as non-Hispanic Black had lower pCR rates than those who were non-Hispanic White.^8^ Black women have also been reported to have a higher frequency of triple-negative breast cancers (TNBCs), ie, breast cancers that are negative for estrogen receptors (ER) and progesterone receptors (PR) and without amplification of erb-b2 receptor tyrosine kinase 2 (*ERBB2*; formerly *HER2* or *HER2/neu*).^9,10^ The variation of pCR rate by tumor subtype^1^ could partially explain racial and ethnic disparities in response to NACT,^11^ but reasons for racial disparities within specific tumor subtypes remain poorly understood. There is a well-documented survival gap between racial and ethnic groups of women with breast cancer for complex reasons, such as access to quality care and biologic heterogeneity.^12,13,14^ With the increasing use of NACT in the US, it is important to know the long-term survival implications of racial and ethnic disparity in pCR to close the widening mortality gap between Black and White patients.

In this study, we sought to evaluate whether there were any racial or ethnic differences in the rate of pCR following NACT in a contemporary National Cancer Database (NCDB) cohort and, if so, whether the racial and ethnic differences in response to therapy varied by molecular subtype. Additionally, we sought to explore the potential factors associated with disparity in pCR, including treatment regimen–related differences, access to care limitations, treatment delays, and granular tumor heterogeneity. We also sought to evaluate whether there was any difference in survival regarding pCR rate achieved by different racial and ethnic groups.

## Methods

### Data Source and Study Sample

The NCDB, jointly sponsored by the American College of Surgeons and the American Cancer Society, is a nationwide, facility-based, oncology data set that captures approximately 70% of all newly diagnosed breast cancer cases in the US.^15,16^ Using the NCDB data from January 2010 to December 2017, we identified patients with stage I to III breast cancer who underwent surgery and received NACT. We excluded patients with clinical T0 disease, those who underwent neoadjuvant radiotherapy, and those without data in pathologic response to chemotherapy. Given our interest in race and ethnicity and the response to NACT, patients without race and ethnicity information were excluded and American Indian and Alaska Native individuals were excluded due to the relatively small sample sizes. Furthermore, we excluded patients who were lost to follow-up (no vital status or <6 months of follow-up) in the survival analysis (eFigure 1 in Supplement 1). The institutional review board at the University of Chicago granted a waiver of informed consent for this study because no protected health information was reviewed and the analysis was retrospective using deidentified data. The study followed the Strengthening the Reporting of Observational Studies in Epidemiology (STROBE) reporting guideline.

### Variables

Patient characteristics were recorded from the NCDB, including age at diagnosis, sex, insurance status, race and ethnicity, clinical and pathologic category (T, N, M), tumor subtype (hormone receptor–positive [HR+]/*ERBB2-*negative [*ERBB2*−], HR+/*ERBB2*+, HR−/*ERBB2*+, or TNBC), tumor grade, histologic type, and year of diagnosis. Race and ethnicity was extracted by cancer registrars from various sources, with high priority given to self-reported race and ethnicity. Information regarding location and treatment details was captured. For treating facility, we included the census region of the facility and facility type. For treatment details, the duration from diagnosis to initiation of chemotherapy and duration of chemotherapy were calculated. Charlson-Deyo comorbidity index^17^ status was calculated from 15 comorbid conditions, such as myocardial infarction and diabetes, using reported *International Classification of Diseases, Ninth Revision, Clinical Modification* or *International Statistical Classification of Diseases and Related Health Problems, 10th Revision* secondary diagnosis codes in cancer registries. In patients with *ERBB2*+ breast cancer, the continuous value of the *ERBB2* to chromosome enumeration probe 17 (CEP17) copy number ratio was also recorded. The primary end point of the analysis, pCR, was defined as the absence of residual invasive cancer on resected breast specimen and lymph nodes after NACT (ypT0/Tis ypN0). In particular, pathologic T category (primary tumor) and N category (regional lymph nodes) post-NACT and on surgical specimen were available in the NCDB; patients with both ypT0 or ypTis (ductal carcinoma in situ) and ypN0 were considered as having a pCR, while patients with other pathologic T and N categories were considered as having a residual disease. Patients with distant metastatic disease were excluded from the analysis. The secondary end point was overall survival, defined as the time from date of diagnosis to date of death or last follow-up.

### Statistical Analysis

Data analysis was conducted from August 2021 to January 2023. Demographic and clinical features were compared among 4 racial and ethnic groups included in this study, as categorized by the NCDB (Asian or Pacific Islander, Hispanic, non-Hispanic Black, and non-Hispanic White; we combined several Asian groups due to small sample size), using χ^2^ tests for categorical variables and linear regressions for continuous variables. Multivariable logistic regression was used to model odds of pCR as a function of racial and ethnic groups and other clinical and pathologic factors. Separate logistic regression models were fit for each molecular subtype to examine in which subtype racial and ethnic disparities existed. Adjusted odds ratios (aORs) and 95% CIs were estimated from logistic regressions. In patients with HR+/*ERBB2*+ and HR−/*ERBB2*+ subtypes, we examined whether the *ERBB2*/CEP17 ratio was associated with pCR. Then, we estimated what proportion of racial and ethnic difference in pCR was mediated by *ERBB2*/CEP17 ratio using the paramed module in the Stata, version 15 package (StataCorp LLC).

Kaplan-Meier curves of overall survival were used to compare survival differences among racial and ethnic groups, stratified by molecular subtype. Mediation analysis was conducted to assess the extent to which racial and ethnic differences in overall survival could be explained by disparity in the pCR rate. We used the general mediation approach proposed by Imai et al,^18^ which can accommodate nonlinear associations and is implemented in the R mediation package.^19^ In particular, we estimated the association between race and ethnicity and overall survival using a Weibull accelerated failure time model and the association between race and ethnicity and pCR using a logistic regression model. Then, we estimated the proportion mediated by pCR. We checked the proportional hazards assumption in survival analysis; if there was serious violation (ie, if hazard curves overlapped) of the assumption, we stratified follow-up time into intervals such that the proportional hazards assumption held in each interval. A 2-sided *P* < .05 was considered statistically significant.

## Results

### Study Sample

The study included 107 207 patients (106 587 [99.4%] women; 620 [0.6%] men) with stage I to III breast cancer, with a mean (SD) age of 53.4 (12.1) years. Of these, 5009 were Asian or Pacific Islander, 18 417 were Black, 9724 were Hispanic, and 74 057 were White. White patients tended to be older at the time of diagnosis compared with the other racial and ethnic groups. Additionally, White and Asian and Pacific Islander patients were more often privately insured, while Black and Hispanic patients were more often enrolled in Medicaid (eTable 1 in Supplement 1).

### Cancer Presentation

Patients across all racial and ethnic categories most commonly presented with T2 tumors at the time of breast cancer diagnosis. White and Asian and Pacific Islander patients were more likely to present with N0 disease, while Black and Hispanic patients presented with N1 disease more commonly than the other racial and ethnic groups. Black patients most often presented with TNBC compared with the other groups (38.9% vs <27%), and Asian and Pacific Islander patients most likely presented with *ERBB2*+ subtypes. All cohorts had a predominance of high-grade disease, but the proportion in Black patients was highest at 68.8% (eTable 1 in Supplement 1).

### Care Context and Therapy Details

White patients were less likely to receive care at an academic or research program (30.0%) compared with the other cohorts (≥41%). Black and Hispanic patients had a longer duration from diagnosis to initiation of NACT compared with White and Asian and Pacific Islander patients (eFigure 2A in Supplement 1). Most patients initiated NACT within 2 months after diagnosis, but 15.6% of Black patients and 20.1% Hispanic patients initiated chemotherapy more than 2 months after diagnosis (eTable 1 in Supplement 1). Similarly, Black and Hispanic patients received NACT approximately 1 week longer than White and Asian and Pacific Islander patients (eTable 1 in Supplement 1). Black and Hispanic patients also had a larger variation in duration of NACT than White and Asian and Pacific Islander patients (eFigure 2B in Supplement 1).

### Pathologic Complete Response

The overall rate of pCR in this cohort was 25.6%. After adjusting for prognostic factors in multivariable logistic regression, Black patients had an overall 9% lower odds of pCR than White patients, while Hispanic patients had 9% higher odds of pCR than White patients (Table 1). Patients with HR+/*ERBB2*− subtype were least likely to achieve pCR (pCR rate, 9.5%) and those with HR−/*ERBB2*+ were most likely to achieve pCR (pCR rate, 51.7%). The pCR rates were 32.6% for HR+/*ERBB2*+ and 30.6% for TNBC subtype. Higher grade, lower clinical T and N category, younger age at diagnosis, female sex, private insurance status, and lower Charlson-Deyo comorbidity index score were all associated with a higher likelihood of achieving pCR. Additionally, patients with less than 31 days from diagnosis to initiation of NACT were more likely to achieve pCR than those experiencing a longer delay to initiation of treatment.

**Table 1.** Pathologic Complete Response According to Demographic and Clinical Factors: Multivariable Logistic Regression

Factor	No. of patients	pCR, No. (%)	aOR (95% CI)^a^	χ^2^^b^
Race and ethnicity				
Asian and Pacific Islander	5009	1406 (28.1)	1.01 (0.94-1.09)	33.3
Black	18 417	4519 (24.5)	0.91 (0.87-0.95)	
Hispanic	9724	2615 (26.9)	1.09 (1.03-1.15)	
White	74 057	18 855 (25.5)	1 [Reference]	
Age at diagnosis, y				
<40	14 722	4490 (30.5)	1.36 (1.25-1.49)	197.1
40-49	26 455	7262 (27.5)	1.09 (1.05-1.14)	
50-59	32 066	8497 (26.5)	1 [Reference]	
60-69	23 858	5290 (22.2)	0.86 (0.82-0.90)	
70-79	8638	1646 (19.1)	0.75 (0.69-0.81)	
≥80	1468	210 (14.3)	0.54 (0.46-0.64)	
Sex				
Female	106 587	27 327 (25.6)	1 [Reference]	26.1
Male	620	68 (11.0)	0.48 (0.37-0.64)	
Insurance status				
None	3868	841 (21.7)	0.82 (0.75-0.90)	57.6
Private	66 664	18 611 (27.9)	1 [Reference]	
Medicaid	12 638	2977 (23.6)	0.87 (0.83-0.92)	
Medicare	20 945	4161 (19.9)	0.86 (0.82-0.91)	
Other government	1395	369 (26.5)	0.97 (0.84-1.11)	
Unknown	1697	436 (25.7)	0.95 (0.82-1.09)	
Charlson-Deyo comorbidity index				
0	93 100	24 203 (26.0)	1 [Reference]	18.3
1	11 323	2639 (23.3)	0.96 (0.91-1.01)	
≥2	2784	553 (19.9)	0.80 (0.71-0.89)	
Subtype				
HR+/*ERBB2*−	38 777	3677 (9.5)	1 [Reference]	6009.1
HR+/*ERBB2*+	23 651	7705 (32.6)	3.62 (3.46-3.80)	
HR−/ *ERBB2*+	12 243	6329 (51.7)	7.91 (7.50-8.35)	
TNBC	29 574	9043 (30.6)	2.87 (2.74-3.00)	
Grade				
Low	5783	477 (8.2)	1 [Reference]	1180.4
Intermediate	35 842	6054 (16.9)	1.64 (1.48-1.82)	
High	61 458	19 308 (31.4)	2.94 (2.65-3.27)	
Histologic type				
Ductal	88 581	24 486 (27.6)	1 [Reference]	371.8
Lobular	5795	462 (8.0)	0.54 (0.48-0.60)	
Ductal and lobular	3349	389 (11.6)	0.60 (0.53-0.68)	
Mucinous	492	22 (4.5)	0.26 (0.16-0.40)	
Papillary	258	61 (23.6)	0.99 (0.70-1.39)	
Inflammatory	5458	1060 (19.4)	1.06 (0.96-1.18)	
Metaplasia	936	105 (11.2)	0.28 (0.22-0.35)	
Other	2338	810 (34.6)	1.37 (1.23-1.52)	
Clinical N category				
N0	52 338	15 955 (30.5)	1 [Reference]	566.3
N1-3	54 138	11 348 (21.0)	0.67 (0.65-0.69)	
Clinical T category				
T1	19 556	6015 (30.8)	1 [Reference]	371.8
T2	53 915	15 117 (28.0)	0.83 (0.80-0.87)	
T3	20 158	3961 (19.6)	0.64 (0.61-0.68)	
T4	13 165	2238 (17.0)	0.53 (0.49-0.57)	
Facility type				
Community cancer program	5753	1156 (20.1)	1 [Reference]	40.3
Comprehensive community cancer program	35 212	8432 (23.9)	1.13 (1.04-1.22)	
Integrated network cancer program	20 380	5206 (25.5)	1.15 (1.06-1.25)	
Academic or research program	31 140	8111 (26.0)	1.24 (1.15-1.34)	
Days from diagnosis to initiation of neoadjuvant chemotherapy				
<31	51 524	14 006 (27.2)	1 [Reference]	54.7
31-60	43 167	10 814 (25.1)	0.91 (0.88-0.94)	
61-90	7690	1633 (21.2)	0.86 (0.80-0.92)	
>90	3118	534 (17.1)	0.76 (0.68-0.85)	
Duration of neoadjuvant chemotherapy, wk				
<13	6302	703 (11.2)	0.37 (0.34-0.41)	684.2
13-17	13 304	2428 (18.3)	0.65 (0.62-0.69)	
18-28	76 580	21 751 (28.4)	1 [Reference]	
29-32	6042	1435 (23.8)	0.95 (0.89-1.02)	
>32	3270	669 (20.5)	0.83 (0.75-0.91)	
Year of diagnosis, per year increase	NA	NA	1.08 (1.07-1.09)	390.4

Abbreviations: aOR, adjusted odds ratio; *ERBB2*, erb-b2 receptor tyrosine kinase 2; HR, hormone receptor; NA, not applicable; −, negative; pCR, pathologic complete response; +, positive; TNBC, triple-negative breast cancer.

^a^ Calculated from multivariable logistic regression estimating pCR, adjusting for all other variables in the table.

^b^ Test for the difference between pCR and non-pCR groups in the multivariable logistic regression. All *P* < .001, so χ^2^ is a better indicator of significance strength.

### pCR and Race and Ethnicity by Subtype

Stratifying by molecular subtype, we found that pCR rate was similar across racial and ethnic groups for HR+/*ERBB2*+ disease (Table 2). Among patients with HR+/*ERBB2*− cancer, Black patients had the highest pCR rate (11.3%), compared with other racial and ethnic groups (all ≤10%), but these differences were no longer statistically significant after adjusting for clinical factors (model 2). Tumor grade was the main mediator of the association between race and pCR. The aOR comparing Black with White patients was 1.09 (95% CI, 0.99-1.20) after adjusting for tumor grade alone. Among patients with HR+/*ERBB2*−cancer, Black individuals had 50.6% high-grade tumors, while White individuals had 39.1% high-grade tumors. Formal mediation analysis estimated tumor grade to be a factor in 61% of the pCR difference between Black and White patients with HR+/*ERBB2*− cancer.

**Table 2.** Pathologic Complete Response by Molecular Subtype and Race and Ethnicity

Subtype and race and ethnicity	No. of patients (% of pCR)	Unadjusted model 1		Multivariable model 2^a^		Multivariable model 3^b^	
		OR (95% CI)	*P* value	aOR (95% CI)	*P* value	aOR (95% CI)	*P* value
**HR**+**/*ERBB2***−							
Asian and Pacific Islander	1765 (10.0)	1.13 (0.96-1.32)	.15	0.94 (0.79-1.12)	.50	0.95 (0.80-1.14)	.59
Black	5927 (11.3)	1.29 (1.18-1.41)	<.001	1.08 (0.98-1.19)	.14	1.14 (1.03-1.27)	.01
Hispanic	3689 (9.9)	1.11 (0.99-1.25)	.08	0.95 (0.84-1.08)	.47	1.03 (0.90-1.18)	.68
White	27 396 (9.0)	1 [Reference]	NA	1 [Reference]	NA	1 [Reference]	NA
**HR**+**/*ERBB2***+							
Asian and Pacific Islander	1324 (34.7)	1.10 (0.98-1.24)	.10	1.00 (0.88-1.13)	.98	0.99 (0.87-1.13)	.91
Black	3074 (31.2)	0.94 (0.87-1.02)	.17	0.93 (0.85-1.02)	.11	0.97 (0.89-1.07)	.58
Hispanic	2092 (34.2)	1.08 (0.98-1.19)	.12	1.02 (0.92-1.12)	.76	1.06 (0.95-1.18)	.30
White	17 161 (32.5)	1 [Reference]	NA	1 [Reference]	NA	1 [Reference]	NA
**HR**−**/*ERBB2***+							
Asian and Pacific Islander	748 (56.8)	1.20 (1.03-1.39)	.02	1.17 (1.00-1.37)	.06	1.16 (0.98-1.37)	.09
Black	1917 (44.8)	0.74 (0.67-0.82)	<.001	0.76 (0.68-0.84)	<.001	0.78 (0.70-0.88)	<.001
Hispanic	1112 (55.2)	1.12 (0.99-1.27)	.07	1.12 (0.98-1.28)	.10	1.12 (0.97-1.30)	.12
White	8466 (52.3)	1 [Reference]	NA	1 [Reference]	NA	1 [Reference]	NA
**TNBC**							
Asian and Pacific Islander	1041 (30.2)	0.94 (0.82-1.08)	.39	0.89 (0.77-1.03)	.12	0.90 (0.77-1.04)	.15
Black	6953 (27.3)	0.82 (0.77-0.87)	<.001	0.82 (0.77-0.87)	<.001	0.85 (0.79-0.91)	<.001
Hispanic	2507 (33.3)	1.09 (1.00-1.19)	.06	1.02 (0.93-1.12)	.64	1.10 (0.99-1.22)	.06
White	19 073 (31.4)	1 [Reference]	NA	1 [Reference]	NA	1 [Reference]	NA

Abbreviations: aOR, adjusted odds ratio; *ERBB2*, erb-b2 receptor tyrosine kinase 2; HR, hormone receptor; NA, not applicable; −, negative; OR, odds ratio; pCR, pathologic complete response; +, positive; TNBC, triple-negative breast cancer.

^a^ Adjusted for year of diagnosis, age, sex, clinical T category, clinical N category, grade, histologic type, and comorbidity index in multivariable logistic regression model 2.

^b^ In addition to variables in model 2, further adjusted for insurance status, facility type, geographic location of facility, days from diagnosis to neoadjuvant chemotherapy, and duration of neoadjuvant chemotherapy.

In HR−/*ERBB2*+ subtype, Asian and Pacific Islander patients achieved the highest pCR rate (56.8%), followed by Hispanic (55.2%) and White (52.3%) patients, with the lowest pCR rate seen in Black patients (44.8%). After adjusting for multiple clinical and sociodemographic factors, Black patients with HR−/*ERBB2*+ disease still had a 22% lower odds of pCR than White patients (aOR, 0.78; 95% CI, 0.70-0.88; *P* < .001). In TNBC subtype, Black patients had a lower pCR rate (27.3%) than other racial and ethnic groups (all >30%). The difference between Black and White patients was significant after adjusting for multiple clinical and sociodemographic factors (aOR, 0.85; 95% CI, 0.79-0.91; *P* < .001).

### *ERBB2*/CEP17 Ratio and pCR Rate

**Figure 1.**
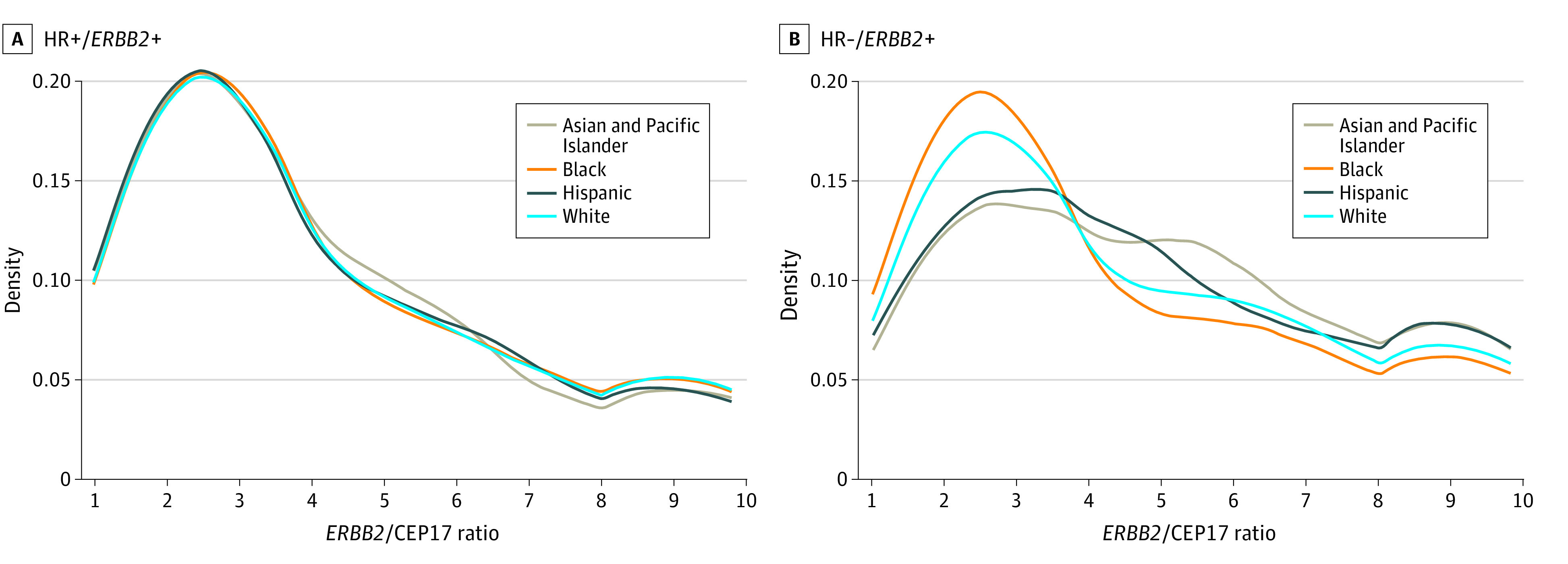
Distribution of Erb-b2 Receptor Tyrosine Kinase 2 (*ERBB2*)/CEP17 Ratio by Race and Ethnicity in Patients With *ERBB2-*Positive (*ERBB2*+) Breast Cancer In the hormone receptor–positive (HR+)/*ERBB2*+ subtype (A), the median *ERBB2*/CEP17 ratio was 3.3 for Asian and Pacific Islander, 3.3 for Black, 3.2 for Hispanic, and 3.3 for White patients. In the hormone receptor–negative (HR−)/*ERBB2*+ subtype (B), the median *ERBB2*/CEP17 ratio was 4.9 for Asian and Pacific Islander, 3.5 for Black, 4.5 for Hispanic, and 4.1 for White patients.

In the HR+/*ERBB2*+ and HR−/*ERBB2*+ subtypes, approximately 38% of patients had available data on the *ERBB2*/CEP17 ratio. We found that, for a 1-unit increase in *ERBB2*/CEP17 ratio, the OR of pCR was 1.20 (95% CI, 1.19-1.22; *P* < .001). In the HR+/*ERBB2*+ subtype, there were no significant differences in the *ERBB2*/CEP17 ratio across race and ethnicity (Figure 1A). By contrast, in the HR−/*ERBB2*+ subtype, Black patients had a slightly but significantly lower *ERBB2*/CEP17 ratio than White patients, and Asian and Pacific Islander patients had a significantly higher *ERBB2*/CEP17 ratio than White patients (Figure 1B). Therefore, it is possible that the racial and ethnic difference in pCR in HR−/*ERBB2*+ subtype could be explained by the racial and ethnic difference in *ERBB2*/CEP17 ratio. To address this hypothesis, we conducted a formal mediation analysis. It estimated that the *ERBB2*/CEP17 ratio mediated 21% of the pCR difference between Asian and Pacific Islander and White patients with HR−/*ERBB2*+, 14% of the pCR difference between Black and White patients with HR−/*ERBB2*+, and 21% of the pCR difference between Black and Asian and Pacific Islander patients with HR−/*ERBB2*+.

### pCR and Overall Survival

**Figure 2.**
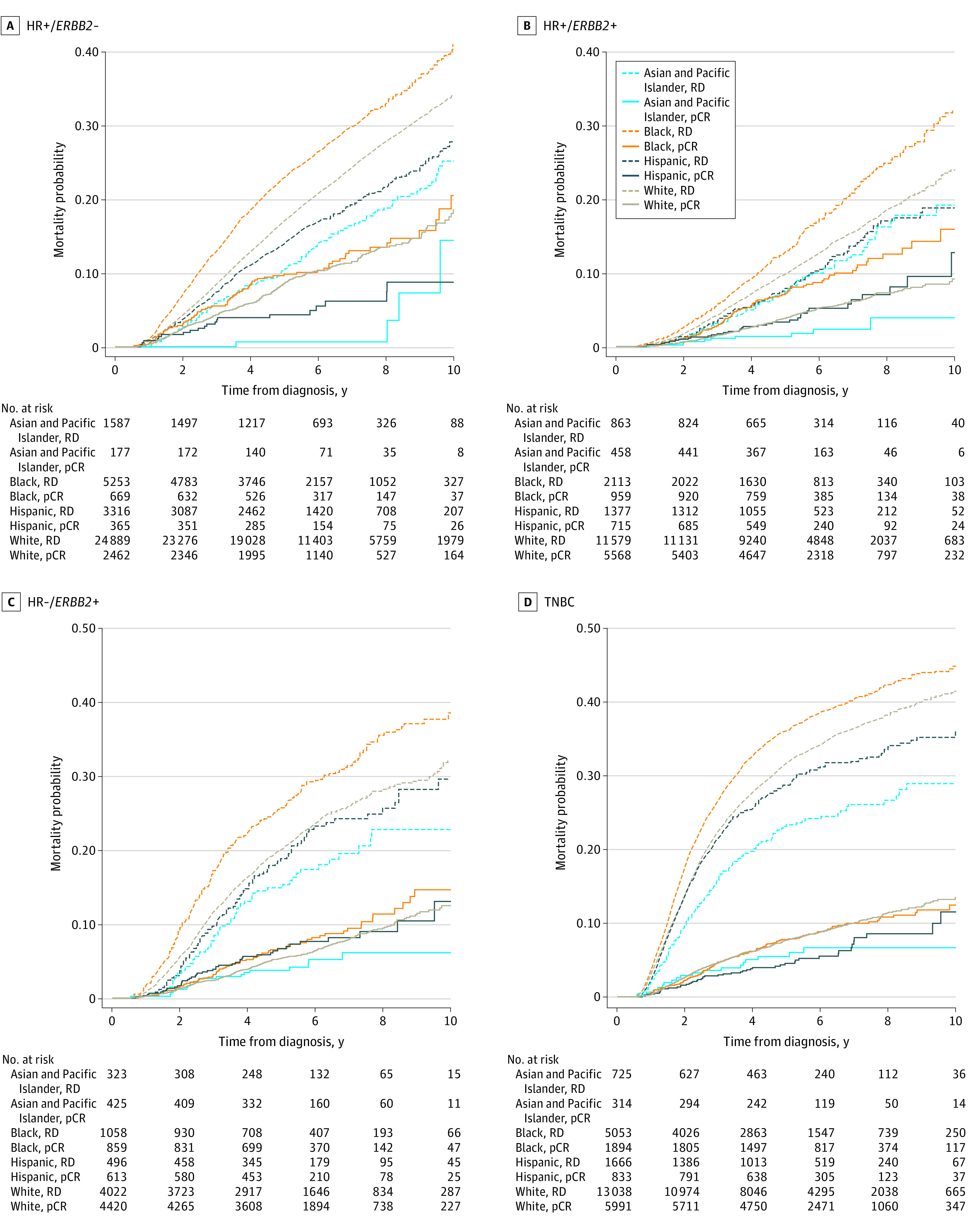
Overall Survival by Race and Ethnicity and Response Status in 4 Molecular Subtypes *ERBB2* indicates erb-b2 receptor tyrosine kinase 2; HR, hormone receptor; −, negative; pCR, pathologic complete response (solid lines); +, positive; RD, residual disease (dashed lines); and TNBC, triple-negative breast cancer.

After a median follow-up of 5.8 years, 21 092 of 107 040 patients died. Achieving pCR after NACT was a significant prognostic factor for overall survival, with a 5-year survival rate of 94% in the pCR cohort compared with 80% in those with residual disease (adjusted hazard ratio [aHR] comparing residual disease vs pCR, 3.45; 95% CI, 3.27-3.63) (eTable 2 in Supplement 1). There were significant disparities in overall survival across racial and ethnic groups in all 4 subtypes among patients with residual disease, with Black patients having the highest mortality risk (Figure 2). In patients achieving pCR, the racial and ethnic difference was small for patients with HR−/*ERBB2*+ or TNBC. For patients with HR+/*ERBB2*+ disease and achieving pCR, Black patients had the lowest survival (Figure 2).

There was a significant disparity in overall survival between Black and White patients with HR−/*ERBB2*+ disease (aHR, 1.35; 95% CI, 1.19-1.53). Formal mediation analysis estimated that the difference in pCR rates between Black and White patients mediated 20% of this survival disparity (Table 3). Similarly, there was a survival difference between Black and Asian and Pacific Islander patients with HR−/*ERBB2*+disease (aHR, 1.73; 95% CI, 1.31-2.29), and 21% of that difference was explained by the difference in pCR rates. In addition, in patients with TNBC, there was a survival disparity between Black and White patients, and 3% of the disparity was mediated by their difference in pCR rates. In HR+/*ERBB2*− subtype, Black patients were less likely to receive endocrine therapy (85.0%) compared with White patients (89.5%) (*P* < .001). The mediation analysis showed that receipt of endocrine therapy could explain 16% of the survival difference between Black and White patients with HR+/*ERBB2*− subtype.

**Table 3.** Mediation Analysis of Race and Ethnicity, Response to Neoadjuvant Chemotherapy, and Overall Survival

Variable	Overall survival, hazard ratio (95% CI)^a^	pCR, odds ratio (95% CI)^b^	Proportion mediated (95% CI)^c^
**In all subtypes^d^**			
Black vs White	1.17 (1.13-1.21)	0.88 (0.84-0.92)	0.17 (0.10-0.24)
**In HR**−**/*ERBB2***+ **subtype**			
Black vs White	1.35 (1.19-1.53)	0.76 (0.68-0.84)	0.20 (0.11-0.35)
Black vs Asian and Pacific Islander	1.73 (1.31-2.29)	0.63 (0.53-0.75)	0.21 (0.11-0.42)
**In TNBC subtype**			
Black vs White	1.09 (1.03-1.16)	0.82 (0.77-0.87)	0.53 (0.34-0.94)

Abbreviations: *ERBB2*, erb-b2 receptor tyrosine kinase 2; HR, hormone receptor; −, negative; pCR, pathologic complete response; +, positive; TNBC, triple-negative breast cancer.

^a^ Hazard ratio adjusted for age, clinical T category, tumor size, clinical N category, grade, histologic type, comorbidity index, and geographic location of cancer center facility in Weibull accelerated failure time model.

^b^ Odds ratio adjusted for age, clinical T category, clinical N category, grade, histologic type, and comorbidity index in logistic regression model.

^c^ Proportion of the association between race and ethnicity with overall survival mediated by its association with pCR.

^d^ In the models for all patients, subtype was also adjusted for.

## Discussion

In our analysis of patients undergoing NACT for breast cancer in this large NCDB cohort, we observed racial and ethnic disparities in response to NACT that were subtype-specific. Additionally, we found that tumor grade was the factor accounting for the greatest level of racial and ethnic disparities in pCR rates among patients with HR+/*ERBB2*− subtype, and the *ERBB2*/CEP17 ratio was an important factor accounting for racial disparities in pCR rates among patients with HR−/*ERBB2*+ subtype. We also noted an association between pCR and overall survival and a significant racial and ethnic disparity in overall survival among patients undergoing NACT. Approximately 20% to 53% of this disparity in overall survival could have been mediated by differences in achieving pCR rates.

These data are consistent with previous literature on variation in pCR by subtype and the resulting association with oncologic outcomes but, to our knowledge, this study represents the largest cohort and most granular analyses to date.^1,20^ In a pooled analysis of 12 clinical trials, subtype-specific pCR rates were 7.5% for HR+/*ERBB2*− grades 1/2 subtype, 12.3% for HR+/*ERBB2*− grade 3 subtype, 30.9% for HR+/*ERBB2*+ subtype, 50.3% for HR−/*ERBB2*+ subtype, and 33.6% for TNBC.^1^ These pCR rates were quite similar to those observed in our study of patients in a clinical practice setting using the same definition of pCR: the subtype-specific pCR rates were 9.5% for HR+/*ERBB2*−, 32.6% for HR+/*ERBB2*+, 51.7% for HR−/*ERBB2*+, and 30.6% for TNBC subtype. We found an association between achieving pCR and overall survival with an aHR of 3.45 for residual disease, as compared with the HR of 2.78 in previous clinical trials,^1^ reconfirming that pCR is a valid intermediate end point for NACT. Fayanju and colleagues^21^ examined the survival implication for anatomic extent of pCR using data from patients with T1-3/N0-1 category cancer in the NCDB and found that patients achieving pCR in both the breast and axilla had better overall survival than patients achieving pCR only in either the breast or axilla.

However, several single-institution studies did not find racial and ethnic disparities in pCR rates,^22,23,24^ while registry-based large studies reported that Black patients achieved lower rates of pCR in triple-negative and HR−/*ERBB2*+ breast cancer.^8,11^ With a larger sample size than previous registry-based studies,^8,11^ we confirmed that Black patients had lower pCR rates than White patients with HR−/*ERBB2*+ and TNBC. Moreover, by extending the analysis to other racial and ethnic groups, we found pCR rates to be highest in Asian and Pacific Islander patients for the HR−/*ERBB2*+ subtype, but there were no racial and ethnic disparities for the HR+/*ERBB2*+ subtype. Consistently, in the HR−/*ERBB2*+ subtype, Asian and Pacific Islander patients had the highest *ERBB2*/CEP17 ratio and Black individuals had the lowest ratio, but the *ERBB2*/CEP17 ratio was quite similar across racial and ethnic groups in the HR+/*ERBB2*+ subtype. An increasing *ERBB2*/CEP17 ratio has been reported to correlate with an increased rate of achieving pCR,^25^ as was noted in our analysis. Hence, by using Asian and Pacific Islander ancestry as the referent group in our study, we found disparities for both Black and White patients and noted that the *ERBB2*/CEP17 ratio may be an important biologic factor explaining why Asian and Pacific Islander patients, who are more likely to be diagnosed with HR−/*ERBB2*+ subtype, also had the highest pCR rate. Furthermore, we noted that Black patients had the highest pCR rate in the HR+/*ERBB2*− subtype, and tumor grade was the major factor accounting for this difference because Black patients were more likely to have high tumor grade and respond to NACT compared with other racial groups. Heterogeneity of ER immunohistochemistry staining could be another granular factor contributing to racial and ethnic disparity. Not all ER+ tumors express high levels of ER expression or activation, and a subset of breast tumors exhibits borderline (1%-9% positive cells) or moderately (10%-50% positive cells) positive ER staining. Borderline ER+ tumors may behave the same as ER− tumors and respond favorably to chemotherapy.^26^ Previous studies have found that Black patients were more likely to have borderline and moderately ER+ or frankly PR− tumors than White patients.^27,28^

### Strengths and Limitations

A major strength of this study is the large number of patients of races and ethnicities other than White included from diverse practice locations, allowing us to observe that tumor biologic factors and nature of disease (eg, tumor grade and *ERBB2*/CEP17 ratio) contributed a significant portion of the differential response to NACT by race and ethnicity. However, lower pCR rates in Black patients with aggressive subtypes of breast cancer may also be rooted in how patients of races and ethnicities other than White experience and receive their therapy. In our study, there were notable delays in initiating NACT for Black and Hispanic patients, as well as larger variation in duration of treatment in these groups, suggesting deviation from typical treatment protocols; this finding is consistent with previous studies.^29,30^ Griggs et al^31^ also found that Black women and women with lower levels of educational attainment were more often the recipients of non–guideline-concordant chemotherapy regimens. These differences in care delivery could translate to lower quality of care and completion of optimal chemotherapy dose intensity for Black patients.^30^ However, after adjusting for delays in initiating NACT and duration of NACT, the aORs in our study did not change much and racial and ethnic differences remained (comparing models 2 and 3 in Table 3), suggesting that additional granular biologic factors need to be considered to improve response to NACT in Black patients.

There are several limitations to our study. First, given the registry nature of the NCDB, central pathologic review was not performed to confirm pCR to NACT. We relied on pathologic T and N categories to determine pCR status. Second, although the NCDB records data on status of receiving NACT, there was no detailed information on specific treatment regimens in the NCDB. For example, there were no data on whether a patient with *ERBB2*+ cancer received trastuzumab or pertuzumab. Third, it is unknown whether patients received adjuvant chemotherapy and type of regimens in the NCDB, although adjuvant radiotherapy and endocrine therapy data were available. The response to NACT affects the delivery of adjuvant therapy, so the adjuvant therapy received may be an intermediate variable in the pathway of the association between pCR and survival, and thus should not be adjusted for in the mediation analysis. Nevertheless, adjusting for radiotherapy and endocrine therapy did not alter the main study findings. Fourth, approximately 62% of the patients with *ERBB2*+ cancer had missing values in the *ERBB2*/CEP17 ratio, so patients with these data available might not be representative, which limits the external generalizability of our findings on the *ERBB2*/CEP17 ratio. Fifth, there were no granular data on percentage of ER-positive staining and tumor somatic mutations. In an accompanying single-institution study, we observed that the percentage of ER positivity and genomic alterations in the mitogen-activated protein kinase pathway may in part explain racial and ethnic differences in response to NACT.^32^

## Conclusions

In this cohort study of patients with breast cancer receiving NACT, we noted racial and ethnic differences in the rate of pCR after NACT, and these differences were subtype-specific. We also identified important biologic factors to explain the racial and ethnic disparities in pCR rates as tumor grade for HR+/*ERBB2*− subtype and *ERBB2*/CEP17 ratio for HR−/*ERBB2*+ subtype. Black patients with HR−/*ERBB2*+ and TNBC were less likely to achieve a pCR after standard-of-care NACT. These findings suggest that the inability to achieve a pCR underscores the need to expand access to biomarker-informed clinical trials in diverse academic and community settings.

## Notes

Supplement 1. **eFigure 1.** Study Flow Chart for Identifying the Cohort of Breast Cancer Patients Receiving Neoadjuvant Chemotherapy Between 2010 and 2017 in the National Cancer Database **eFigure 2.** Distribution of Days From Diagnosis to the Initiation of Neoadjuvant Chemotherapy and Duration of Neoadjuvant Chemotherapy, by Race and Ethnicity **eTable 1.** Demographic and Clinical Factors by Race and Ethnicity in Patients Receiving NACT **eTable 2.** Hazard Ratios in the Weibull Accelerated Failure Time Models of Overall Survival by Subtype

Supplement 2.
Data Sharing Statement
