# Supplementary material for: Response to Treatment, Racial and Ethnic Disparity, and Survival in Patients With Breast Cancer Undergoing Neoadjuvant Chemotherapy in the US
Source: JAMA Netw Open. 2023 Mar 30;6(3):e235834. doi: 10.1001/jamanetworkopen.2023.5834 (PMC10064248; doi:10.1001/jamanetworkopen.2023.5834)
Supplement: Supplement 1. — eFigure 1. Study Flow Chart for Identifying the Cohort of Breast Cancer Patients Receiving Neoadjuvant Chemotherapy Between 2010 and 2017 in the National Cancer Database eFigure 2. Distribution of Days From Diagnosis to the Initiation of Neoadjuvant Chemotherapy and Duration of Neoadjuvant Chemotherapy, by Race and Ethnicity eTable 1. Demographic and Clinical Factors by Race and Ethnicity in Patients Receiving NACT eTable 2. Hazard Ratios in the Weibull Accelerated Failure Time Models of Overall Survival by Subtype [file jamanetwopen-e235834-s001.pdf]

Supplementary Online Content

Shubeck S, Zhao F, Howard FM, Olopade OI, Huo D. Response to treatment, racial and ethnic disparity, and survival in patients with breast cancer undergoing neoadjuvant chemotherapy in the US. *JAMA Netw Open*. 2023;6(3):e235834. doi:10.1001/jamanetworkopen.2023.5834

eFigure 1. Study Flow Chart for Identifying the Cohort of Breast Cancer Patients Receiving Neoadjuvant Chemotherapy Between 2010 and 2017 in the National Cancer Database

eFigure 2. Distribution of Days From Diagnosis to the Initiation of Neoadjuvant Chemotherapy and Duration of Neoadjuvant Chemotherapy, by Race and Ethnicity

eTable 1. Demographic and Clinical Factors by Race and Ethnicity in Patients Receiving NACT

eTable 2. Hazard Ratios in the Weibull Accelerated Failure Time Models of Overall Survival by Subtype

This supplementary material has been provided by the authors to give readers additional information about their work.

© 2023 Shubeck S et al. *JAMA Network Open.*

eFigure 1. Study flow chart for identifying the cohort of breast cancer patients receiving neoadjuvant chemotherapy between 2010 and 2017 in the National Cancer Database

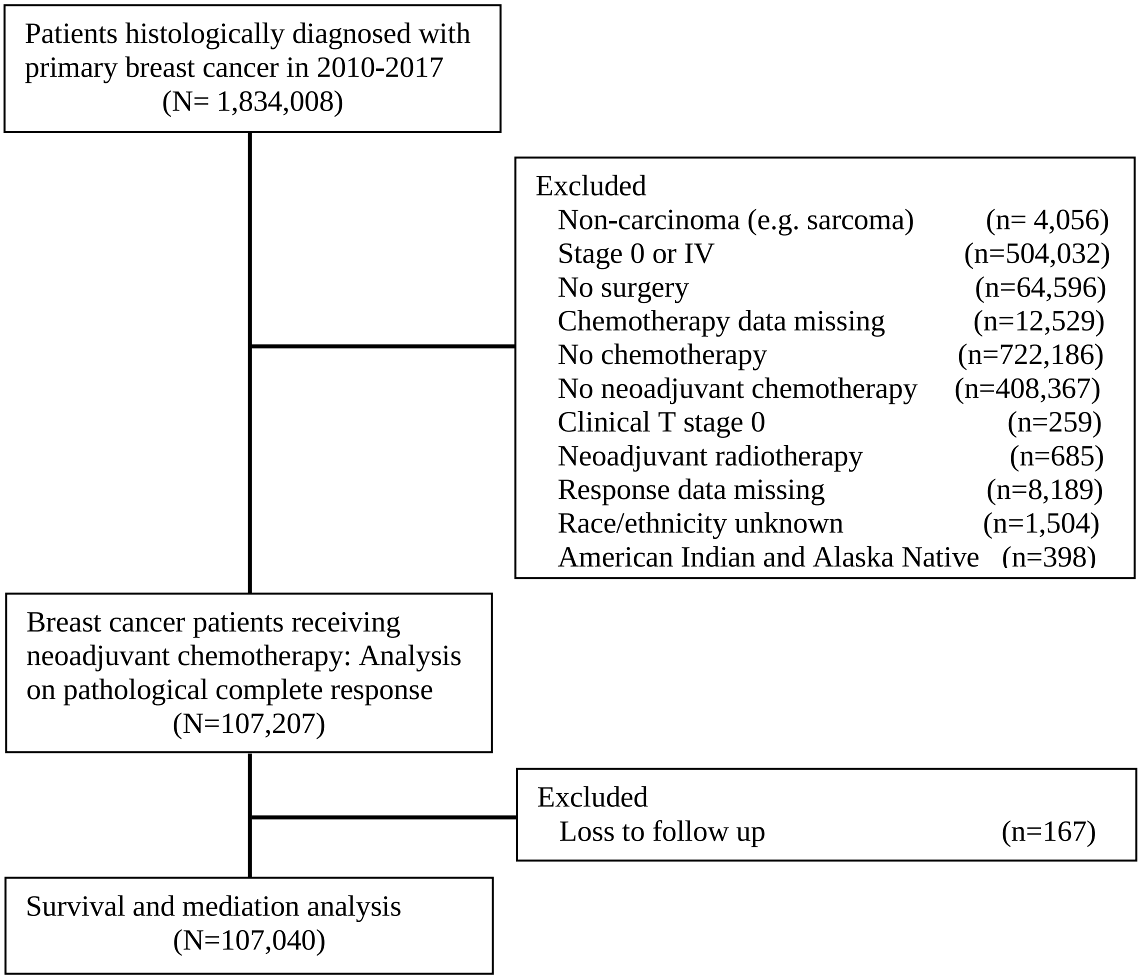

© 2023 Shubeck S et al. *JAMA Network Open.*

eFigure 2. Distribution of days from diagnosis to the initiation of neoadjuvant chemotherapy (A) and duration of neoadjuvant chemotherapy (B), by race and ethnicity. SD, standard deviation

a.

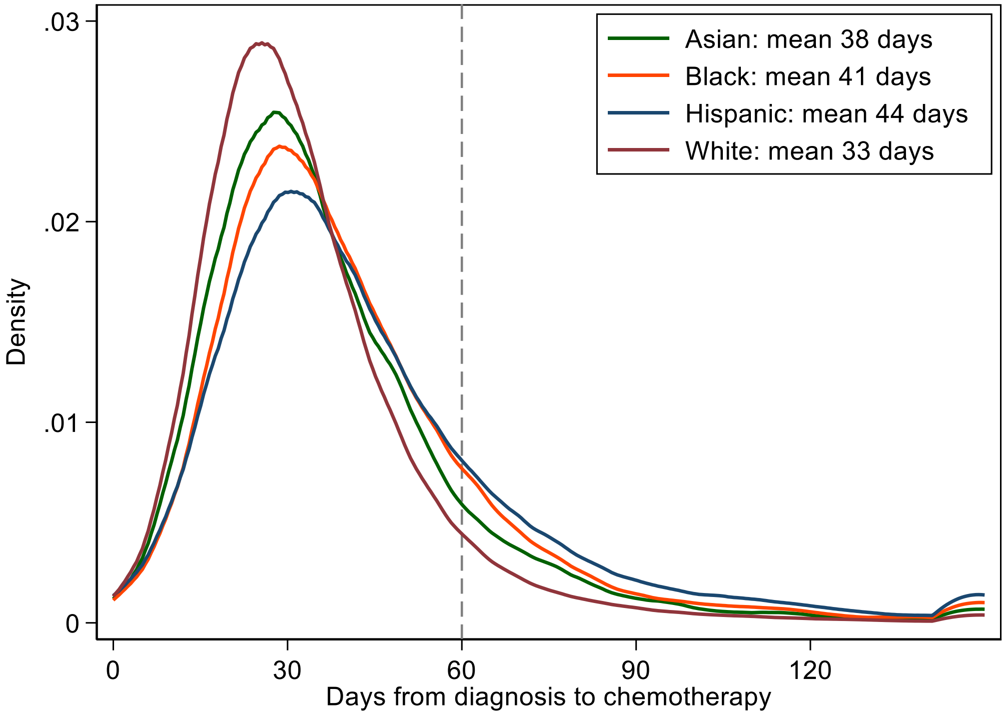

b.

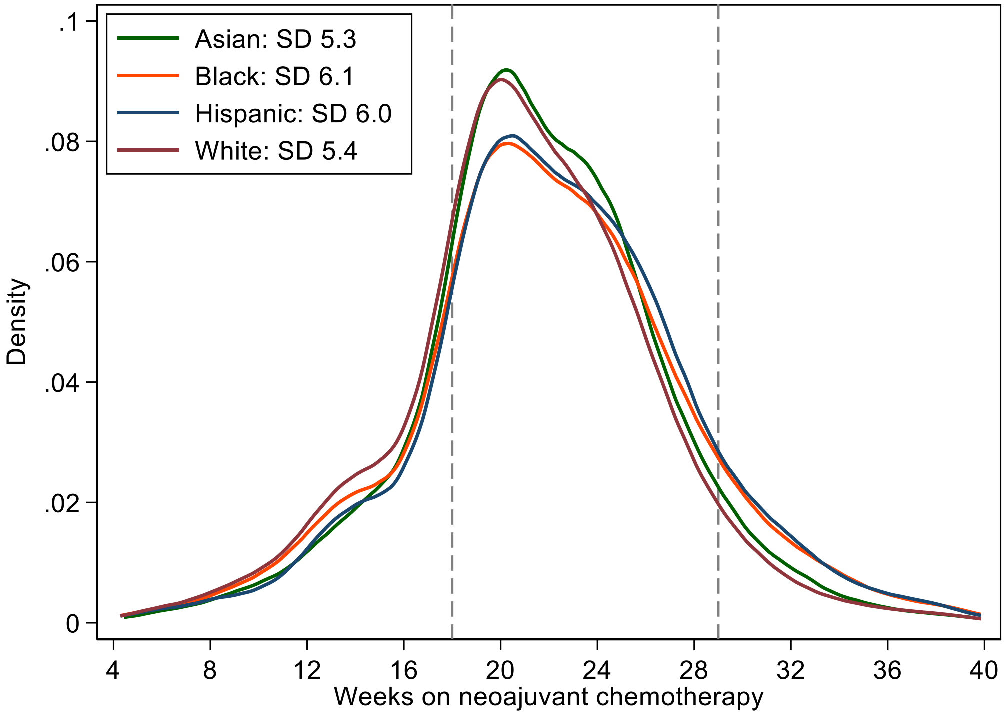

© 2023 Shubeck S et al. *JAMA Network Open.*

eTable 1. Demographic and clinical factors by race and ethnicity in patients receiving NACT

	Asian (N=5009)	Black (N=18417)	Hispanic (N=9724)	White (N=74057)	p-value
	n (%)	n (%)	n (%)	n (%)	
Age at diagnosis					<0.001
<40	915 (18.3)	2662 (14.5)	1957 (20.1)	9188 (12.4)	
40-49	1481 (29.6)	4847 (26.3)	3167 (32.6)	16960 (22.9)	
50-59	1405 (28.0)	5925 (32.2)	2628 (27.0)	22108 (29.9)	
60-69	928 (18.5)	3608 (19.6)	1440 (14.8)	17882 (24.1)	
70-79	243 (4.9)	1203 (6.5)	471 (4.8)	6721 (9.1)	
80+	37 (0.7)	172 (0.9)	61 (0.6)	1198 (1.6)	
mean ± SD	50.6 ± 11.6	52.3 ± 11.7	49.6 ± 11.6	54.3 ± 12.2	<0.001
Sex					<0.001
Male	18 (0.4)	119 (0.6)	33 (0.3)	450 (0.6)	
Female	4991 (99.6)	18298 (99.4)	9691 (99.7)	73607 (99.4)	
Insurance status					<0.001
Not Insured	211 (4.2)	915 (5.0)	1292 (13.3)	1450 (2.0)	
Private Insurance	3361 (67.1)	10024 (54.4)	4455 (45.8)	48824 (65.9)	
Medicaid	766 (15.3)	3356 (18.2)	2550 (26.2)	5966 (8.1)	
Medicare	521 (10.4)	3476 (18.9)	1108 (11.4)	15840 (21.4)	
Other government	48 (1.0)	311 (1.7)	100 (1.0)	936 (1.3)	
Unknown	102 (2.0)	335 (1.8)	219 (2.3)	1041 (1.4)	
Charlson comorbidity index					<0.001
0	4474 (89.3)	15148 (82.3)	8515 (87.6)	64963 (87.7)	
1	460 (9.2)	2550 (13.8)	1030 (10.6)	7283 (9.8)	
2+	75 (1.5)	719 (3.9)	179 (1.8)	1811 (2.4)	
Clinical T stage					<0.001
T1	714 (14.3)	3233 (17.6)	1509 (15.6)	14100 (19.1)	
T2	2842 (57.0)	8927 (48.6)	5007 (51.8)	37139 (50.3)	
T3	927 (18.6)	3790 (20.6)	2086 (21.6)	13355 (18.1)	
T4	504 (10.1)	2407 (13.1)	1058 (11.0)	9196 (12.5)	
Tumor size in cm, mean ± SD	3.8 ± 2.4	3.9 ± 2.8	3.8 ± 2.5	3.6 ± 2.5	<0.001
Clinical N stage					<0.001
N0	2417 (48.6)	7838 (42.8)	4124 (42.8)	37959 (51.6)	
N1	1991 (40.0)	7720 (42.2)	4240 (44.0)	27646 (37.6)	
N2	324 (6.5)	1662 (9.1)	759 (7.9)	4832 (6.6)	
N3	243 (4.9)	1092 (6.0)	506 (5.3)	3123 (4.2)	
Clinical subtype					<0.001
HR+/HER2-	1765 (36.2)	5927 (33.2)	3689 (39.2)	27396 (38.0)	
HR+/HER2+	1324 (27.1)	3074 (17.2)	2092 (22.3)	17161 (23.8)	
HR-/HER2+	748 (15.3)	1917 (10.7)	1112 (11.8)	8466 (11.7)	
TNBC	1041 (21.3)	6953 (38.9)	2507 (26.7)	19073 (26.5)	

© 2023 Shubeck S et al. *JAMA Network Open.*

Grade					<0.001
Low	205 (4.3)	692 (3.9)	456 (4.9)	4430 (6.2)	
Intermediate	1696 (35.3)	4831 (27.3)	3165 (34.0)	26150 (36.7)	
High	2910 (60.5)	12175 (68.8)	5685 (61.1)	40688 (57.1)	
Histological type					<0.001
Ductal	4388 (87.6)	15677 (85.1)	8208 (84.4)	60308 (81.4)	
Lobular	152 (3.0)	642 (3.5)	427 (4.4)	4574 (6.2)	
D&L	147 (2.9)	402 (2.2)	298 (3.1)	2502 (3.4)	
Mucinous	36 (0.7)	111 (0.6)	42 (0.4)	303 (0.4)	
Papillary	10 (0.2)	66 (0.4)	32 (0.3)	150 (0.2)	
Inflammatory	156 (3.1)	879 (4.8)	424 (4.4)	3999 (5.4)	
Metaplasia	20 (0.4)	204 (1.1)	71 (0.7)	641 (0.9)	
Others	100 (2.0)	436 (2.4)	222 (2.3)	1580 (2.1)	
Type of surgery					<0.001
Breast conserving surgery	1742 (34.8)	7234 (39.3)	3357 (34.5)	26025 (35.1)	
Mastectomy	2296 (45.8)	7579 (41.2)	4333 (44.6)	27403 (37.0)	
Bilateral mastectomy	971 (19.4)	3604 (19.6)	2034 (20.9)	20629 (27.9)	
Endocrine therapy					<0.001
No	2060 (41.7)	10079 (55.5)	4282 (44.7)	32090 (43.9)	
Yes, adjuvant	2542 (51.5)	7024 (38.7)	4639 (48.4)	35865 (49.1)	
Yes, neoadjuvant	334 (6.8)	1047 (5.8)	661 (6.9)	5077 (7.0)	
Radiotherapy					<0.001
No	1353 (27.9)	4508 (25.5)	2606 (27.9)	20491 (28.7)	
Yes	3499 (72.1)	13181 (74.5)	6725 (72.1)	50953 (71.3)	
Facility type					<0.001
Community cancer program	167 (4.1)	747 (4.7)	520 (6.7)	4319 (6.7)	
Comprehensive community cancer program	1443 (35.2)	5206 (33.0)	2375 (30.6)	26188 (40.4)	
Integrated network cancer program	763 (18.6)	3342 (21.2)	1361 (17.5)	14914 (23.0)	
Academic/research program	1721 (42.0)	6460 (41.0)	3511 (45.2)	19448 (30.0)	
Facility location					<0.001
New England	119 (2.9)	288 (1.8)	208 (2.7)	3410 (5.3)	
Middle Atlantic	656 (16.0)	2021 (12.8)	961 (12.4)	8276 (12.8)	
South Atlantic	600 (14.7)	5893 (37.4)	1190 (15.3)	12973 (20.0)	
East North Central	374 (9.1)	2440 (15.5)	528 (6.8)	12765 (19.7)	
East South Central	47 (1.1)	1678 (10.7)	59 (0.8)	4314 (6.7)	
West North Central	128 (3.1)	643 (4.1)	126 (1.6)	6367 (9.8)	
West South Central	327 (8.0)	1946 (12.4)	1974 (25.4)	6066 (9.4)	
Mountain	140 (3.4)	167 (1.1)	640 (8.2)	3497 (5.4)	
Pacific	1703 (41.6)	679 (4.3)	2081 (26.8)	7201 (11.1)	
% of no high school degree quartiles 2012-2016					<0.001
>= 17.6	980 (22.4)	5631 (35.7)	4668 (54.9)	8662 (13.6)	

© 2023 Shubeck S et al. *JAMA Network Open.*

10.9-17.5	842 (19.3)	5125 (32.5)	1672 (19.7)	15267 (24.0)	
6.3-10.8	1160 (26.5)	3421 (21.7)	1316 (15.5)	19619 (30.8)	
< 6.3	1390 (31.8)	1583 (10.0)	840 (9.9)	20126 (31.6)	
Median income quartiles 2012-2016					<0.001
< $40,227	308 (7.0)	6117 (38.9)	2316 (27.3)	7516 (11.8)	
$40,227-$50,353	550 (12.6)	3425 (21.8)	1925 (22.7)	12937 (20.4)	
$50,354-$63,332	904 (20.7)	2920 (18.6)	1940 (22.9)	15557 (24.5)	
>= $63,333	2608 (59.7)	3279 (20.8)	2309 (27.2)	27554 (43.3)	
Urban/Rura continuum					<0.001
Metropolitan	4769 (97.3)	16753 (93.0)	9178 (95.6)	61383 (85.3)	
Urban	116 (2.4)	1103 (6.1)	393 (4.1)	9410 (13.1)	
Rural	18 (0.4)	151 (0.8)	27 (0.3)	1144 (1.6)	
Time from diagnosis to initiation of NACT (days)					<0.001
< 31	2181 (44.9)	7033 (38.8)	3343 (35.3)	38967 (53.4)	
31-60	2084 (42.9)	8264 (45.5)	4220 (44.6)	28599 (39.2)	
61-90	425 (8.7)	2005 (11.0)	1247 (13.2)	4013 (5.5)	
> 90	170 (3.5)	843 (4.6)	650 (6.9)	1455 (2.0)	
mean ± SD	37.8 ± 23.0	41.2 ± 24.8	44.2 ± 27.7	33.5 ± 19.6	<0.001
Duration on NACT (weeks)					<0.001
< 13	222 (4.6)	1045 (5.8)	437 (4.6)	4598 (6.3)	
13-17	548 (11.3)	2058 (11.3)	984 (10.4)	9714 (13.3)	
18-28	3675 (75.6)	12718 (70.1)	6809 (72.0)	53378 (73.1)	
29-32	285 (5.9)	1387 (7.6)	757 (8.0)	3613 (4.9)	
> 32	130 (2.7)	936 (5.2)	473 (5.0)	1731 (2.4)	
mean ± SD	21.8 ± 5.3	22.3 ± 6.1	22.6 ± 6.0	21.2 ± 5.4	<0.001
Year of diagnosis					<0.001
2010	325 (6.5)	1525 (8.3)	695 (7.1)	5965 (8.1)	
2011	413 (8.2)	1694 (9.2)	866 (8.9)	6691 (9.0)	
2012	432 (8.6)	1833 (10.0)	906 (9.3)	6945 (9.4)	
2013	489 (9.8)	2137 (11.6)	1108 (11.4)	7966 (10.8)	
2014	702 (14.0)	2478 (13.5)	1299 (13.4)	10266 (13.9)	
2015	774 (15.5)	2777 (15.1)	1450 (14.9)	11491 (15.5)	
2016	895 (17.9)	3007 (16.3)	1592 (16.4)	12273 (16.6)	
2017	979 (19.5)	2966 (16.1)	1808 (18.6)	12460 (16.8)	

Abbreviations: NACT, neoadjuvant chemotherapy; SD, standard deviation; HR, hormone receptor; HER2, epidermal growth factor receptor 2; TNBC, triple-negative breast cancer.

© 2023 Shubeck S et al. *JAMA Network Open.*

eTable 2. Hazard ratios in the Weibull accelerated failure time models of overall survival by subtype

	All subtypes	HR+/HER2-	HR+/HER2+	HR-/HER2+	TNBC
Residual disease vs. pCR	**3.45 (3.27-3.63)**	**2.71 (2.41-3.05)**	**2.49 (2.21-2.82)**	**3.48 (3.08-3.93)**	**4.12 (3.80-4.47)**
Race/ethnicity					
Asian	0.71 (0.65-0.78)	0.69 (0.60-0.79)	0.87 (0.69-1.09)	0.78 (0.59-1.03)	0.68 (0.58-0.80)
Black	**1.14 (1.10-1.19)**	**1.15 (1.08-1.23)**	**1.30 (1.15-1.46)**	**1.35 (1.19-1.54)**	**1.06 (1.00-1.13)**
Hispanic	0.90 (0.84-0.95)	0.84 (0.77-0.93)	1.07 (0.90-1.26)	1.06 (0.87-1.29)	0.87 (0.79-0.96)
White	1 (ref.)	1 (ref.)	1 (ref.)	1 (ref.)	1 (ref.)
Age at diagnosis					
<40	1.09 (0.99-1.21)	1.30 (1.11-1.53)	1.03 (0.77-1.38)	0.81 (0.58-1.12)	1.03 (0.88-1.21)
40-44	1 (ref.)	1 (ref.)	1 (ref.)	1 (ref.)	1 (ref.)
45-49	0.93 (0.87-1.00)	0.93 (0.84-1.04)	1.02 (0.83-1.26)	0.90 (0.71-1.14)	0.92 (0.83-1.02)
50-54	1.10 (1.03-1.17)	1.22 (1.11-1.35)	1.26 (1.03-1.53)	1.09 (0.88-1.36)	0.94 (0.85-1.04)
55-59	1.18 (1.11-1.26)	1.33 (1.20-1.47)	1.50 (1.24-1.83)	0.99 (0.79-1.24)	1.01 (0.92-1.12)
60-64	1.22 (1.14-1.30)	1.37 (1.23-1.51)	1.42 (1.16-1.74)	1.14 (0.91-1.44)	1.04 (0.94-1.16)
65-69	1.43 (1.34-1.53)	1.60 (1.44-1.77)	2.10 (1.72-2.56)	1.34 (1.05-1.70)	1.18 (1.06-1.31)
70-74	1.74 (1.61-1.87)	1.95 (1.73-2.19)	2.67 (2.15-3.32)	1.90 (1.48-2.45)	1.37 (1.21-1.54)
75-79	2.14 (1.97-2.34)	2.40 (2.09-2.75)	3.46 (2.73-4.38)	2.31 (1.76-3.03)	1.66 (1.45-1.91)
80+	3.24 (2.94-3.56)	3.52 (2.97-4.17)	6.26 (4.96-7.91)	3.50 (2.65-4.62)	2.22 (1.89-2.61)
Clinical N stage					
N0	1 (ref.)	1 (ref.)	1 (ref.)	1 (ref.)	1 (ref.)
N1	1.52 (1.47-1.57)	1.35 (1.28-1.42)	1.56 (1.41-1.72)	1.48 (1.31-1.67)	1.64 (1.55-1.74)
N2	1.92 (1.83-2.03)	1.65 (1.52-1.78)	1.94 (1.66-2.28)	1.85 (1.55-2.21)	2.08 (1.91-2.26)
N3	2.35 (2.21-2.49)	2.05 (1.86-2.25)	2.32 (1.92-2.80)	2.15 (1.77-2.61)	2.60 (2.37-2.85)
Clinical T stage					
T0	1 (ref.)	1 (ref.)	1 (ref.)	1 (ref.)	1 (ref.)
T1	1.03 (0.98-1.08)	1.01 (0.94-1.09)	1.19 (1.04-1.37)	1.00 (0.85-1.18)	1.00 (0.93-1.08)
T2	1.17 (1.10-1.24)	1.03 (0.94-1.13)	1.31 (1.10-1.57)	1.20 (0.98-1.47)	1.24 (1.13-1.36)
T3	1.57 (1.47-1.67)	1.45 (1.32-1.60)	1.93 (1.60-2.32)	1.53 (1.23-1.90)	1.55 (1.39-1.72)
Tumor size, per 1 cm	1.06 (1.05-1.06)	1.06 (1.05-1.07)	1.06 (1.04-1.08)	1.05 (1.03-1.07)	1.06 (1.05-1.07)
Charlson comorbidity index					
0	1 (ref.)	1 (ref.)	1 (ref.)	1 (ref.)	1 (ref.)
1	1.23 (1.18-1.28)	1.25 (1.17-1.34)	1.19 (1.05-1.35)	1.16 (1.00-1.35)	1.23 (1.15-1.32)
2+	1.67 (1.55-1.79)	1.78 (1.60-1.99)	1.98 (1.63-2.40)	2.00 (1.59-2.51)	1.49 (1.32-1.69)
Histological type					
Ductal	1 (ref.)	1 (ref.)	1 (ref.)	1 (ref.)	1 (ref.)
Lobular	1.23 (1.15-1.31)	1.23 (1.14-1.33)	1.40 (1.15-1.70)	1.62 (1.08-2.44)	1.32 (1.08-1.62)
Ductal & lobular	1.24 (1.15-1.35)	1.19 (1.08-1.32)	1.12 (0.89-1.42)	1.46 (0.96-2.21)	1.76 (1.45-2.14)
Others	1.23 (1.17-1.30)	1.20 (1.10-1.30)	1.27 (1.09-1.49)	1.20 (1.01-1.41)	1.25 (1.16-1.35)
Grade					
Low	1 (ref.)	1 (ref.)	1 (ref.)	1 (ref.)	1 (ref.)

© 2023 Shubeck S et al. *JAMA Network Open.*

Intermediate	1.32 (1.22-1.43)	1.33 (1.21-1.45)	0.97 (0.79-1.21)	1.37 (0.86-2.17)	1.45 (1.11-1.91)
High	1.75 (1.62-1.90)	2.01 (1.84-2.20)	1.22 (0.99-1.51)	1.42 (0.90-2.24)	1.57 (1.20-2.06)
Radiotherapy vs. no	0.86 (0.83-0.89)	0.88 (0.83-0.94)	0.84 (0.76-0.92)	0.88 (0.79-0.99)	0.86 (0.81-0.91)
Enhormone endocrine vs. no	0.64 (0.60-0.67)	0.57 (0.54-0.61)	0.70 (0.62-0.78)		
Facility location					
New England	1 (ref.)	1 (ref.)	1 (ref.)	1 (ref.)	1 (ref.)
Middle Atlantic	0.97 (0.89-1.06)	1.01 (0.88-1.17)	0.95 (0.75-1.22)	0.81 (0.61-1.06)	1.03 (0.89-1.19)
South Atlantic	1.11 (1.02-1.20)	1.18 (1.04-1.35)	1.03 (0.82-1.30)	0.87 (0.67-1.12)	1.15 (1.00-1.32)
East North Central	1.09 (1.00-1.19)	1.20 (1.04-1.37)	1.01 (0.80-1.27)	0.98 (0.76-1.27)	1.08 (0.94-1.24)
East South Central	1.19 (1.08-1.31)	1.21 (1.04-1.42)	1.26 (0.97-1.64)	1.00 (0.74-1.35)	1.22 (1.04-1.42)
West North Central	0.96 (0.87-1.05)	1.00 (0.86-1.17)	0.78 (0.60-1.02)	0.82 (0.61-1.11)	1.05 (0.89-1.23)
West South Central	0.92 (0.84-1.01)	1.00 (0.86-1.15)	0.83 (0.64-1.08)	0.80 (0.60-1.07)	0.95 (0.81-1.10)
Mountain	0.99 (0.89-1.10)	1.09 (0.93-1.29)	0.81 (0.60-1.08)	0.94 (0.68-1.30)	1.01 (0.84-1.21)
Pacific	1.00 (0.91-1.09)	1.06 (0.92-1.22)	0.85 (0.67-1.09)	0.76 (0.57-1.01)	1.08 (0.93-1.25)
Subtype, <5 years					
HR+/HER2-	1 (ref.)				
HR+/HER2+	0.58 (0.55-0.62)				
HR-/HER2+	0.71 (0.66-0.77)				
TNBC	1.33 (1.26-1.42)				
Subtype, >=5 years					
HR+/HER2-	1 (ref.)				
HR+/HER2+	0.78 (0.72-0.85)				
HR-/HER2+	0.48 (0.42-0.54)				
TNBC	0.44 (0.40-0.48)				
Shape parameter σ	0.644	0.672	0.584	0.746	0.902

Abbreviations: pCR, pathological complete response; HR, hormone receptor; HER2, epidermal growth factor receptor 2; TNBC, triple-negative breast cancer cer.

© 2023 Shubeck S et al. *JAMA Network Open.*
