# Supplementary material for: Response to Treatment, Racial and Ethnic Disparity, and Survival in Patients With Breast Cancer Undergoing Neoadjuvant Chemotherapy in the US
Source: JAMA Netw Open. 2023 Mar 30;6(3):e235834. doi: 10.1001/jamanetworkopen.2023.5834 (PMC10064248; doi:10.1001/jamanetworkopen.2023.5834)
Supplement: Supplement 2. — Data Sharing Statement [file jamanetwopen-e235834-s002.pdf]

Data Sharing Statement

Shubeck. Response to Treatment, Racial and Ethnic Disparity, and Survival in Patients With Breast Cancer Undergoing Neoadjuvant Chemotherapy in the US. *JAMA Netw Open*. Published March 30, 2023. doi:10.1001/jamanetworkopen.2023.5834

Data
Data available: Yes
Data types: Deidentified participant data
How to access data: The study analyzed data from the National Cancer Database, which collects deidentified data of cancer patients across the United States. Authors are prohibited to share the data per data use agreement, but interested parties could apply for the data directly.
When available: With publication

Supporting Documents
Document types: None

Additional Information
Who can access the data: National Cancer Database.
Types of analyses: For researchers with interest to replicate the study findings, the study author can share analysis codes.
Mechanisms of data availability: After approval of a proposal by National Cancer Database, the data will be make available.
